# The impact of social connectedness on mental health in LGBTQ + identifying individuals during the COVID-19 pandemic in Germany

**DOI:** 10.1186/s40359-023-01265-5

**Published:** 2023-08-29

**Authors:** Christine Firk, Nicola Großheinrich, Norbert Scherbaum, Daniel Deimel

**Affiliations:** 1 Catholic University of Applied Sciences North Rhine–Westphalia, Robert-Schuman- Str. 25, 52066 Aachen, Germany; 2 Institute of Health Research and Social Psychiatry, Catholic University of Applied Sciences North Rhine–Westphalia, Robert-Schuman-Str. 25, 52066 Aachen, Germany; 3 Catholic University of Applied Sciences of North Rhine–Westphalia, Wörthstraße 10, 50668 Cologne, Germany; 4 Department of Psychiatry and Psychotherapy, Medical Faculty, LVR-University Hospital Essen, University of Duisburg-Essen, Essen, Germany; 5 German Institute for Addiction and Prevention Research, Catholic University of Applied Sciences North Rhine–Westphalia, Konrad-Adenauer-Ufer 79-81, 50668 Cologne, Germany

**Keywords:** LGBTQ+; gender identity, Sexual orientation; COVID-19; mental health, Depression, Anxiety, Suicidality, Loneliness, Social isolation

## Abstract

**Background:**

Recent studies report that LGBTQ + people have experienced high levels of mental health problems during COVID-19-related social distancing. Given the well-established association between social isolation and mental health, the main aim of the current study was to investigate differences in mental health and (perceived) social isolation and social support in LGBTQ + individuals compared to heterosexual, cisgender people and to explore whether the hypothesized higher mental health burden in LGBTQ + individuals is (partly) mediated by (perceived) social isolation or social support.

**Methods:**

N = 531 participants indicating belonging to the LGBTQ + community and N = 1826 not identifying as LGBTQ + participated in a cross-sectional online survey during the initial COVID-19-related lockdown in Germany. Standardized questionnaires were used to assess depression, anxiety, suicidality, loneliness and social support. Further, perceived social isolation and face-to-face communication during the lockdown were assessed.

**Results:**

LGBTQ + people had higher levels of depression, anxiety and suicidal thought, were lonelier and experienced less social support than non-LGBTQ + identifying individuals. Mediation analysis showed that the higher levels of mental health burden in LGBTQ + people were (partly) mediated by reduced social connectedness. Further face-to-face contact positively affected mental health by reducing feelings of loneliness.

**Conclusion:**

Given the high impact of loneliness on mental health, governmental actions should be taken to promote social connectedness particularly among LGBTQ + identifying individuals to ensure that the COVID-19 pandemic does not exacerbate the health inequalities that already exist between LGBTQ+-identifying and heterosexual, cisgender people.

## Introduction

The COVID-19 pandemic has had a worldwide impact on the economy, employment, and health but has also been a challenge for individuals’ daily life. Public health and governmental efforts to reduce the spread of the virus have led to a set of actions to reduce the number of face-to-face contacts in many countries. In Germany, during the initial lockdown in the spring of 2020, contact with people outside the household was limited to an absolute minimum. This COVID-19 pandemic required “social distancing” has increased the prevalence of social isolation i.e. the objective lack of, or reduction in, social contacts, and has resulted in higher levels of loneliness i.e. perceived social isolation [1]. A large body of evidence has shown that social isolation and loneliness have long-lasting consequences for physical health [2–4] and have even been associated with a high risk of mortality [5]. Previous studies have also found negative effects of social isolation and loneliness on mental health [6], suicidal behaviors [7] and alcohol consumption [8]. Thus, (perceived) social isolation seems to have devastating effects on human well-being underscoring that social connectedness seems to be a fundamental part of human nature. Therefore, COVID-19-related social isolation and loneliness may have increased mental health disorders, particularly in individuals vulnerable to loneliness and mental health problems [9–11].

LGBTQ + individuals -referring to lesbian, gay, bisexual, transgender, and queer people plus individuals using different terms to describe their sexual orientation or gender identity- compared to the general population, are significantly more likely to report depression, anxiety, suicidal thoughts, and substance use [12–15] and are at increased risk for loneliness [16–18] which has been explained by high levels of minority stress [19, 20]. Minority stress including proximal stressors (i.e. discrimination experiences) and distal stressors (i.e. subjective responses such as self-stigma, or sexual orientation rejection sensitivity) may increase social isolation and decrease mental health by self-protective social withdrawal [21]. During COVID-19-related social distancing, minority stress may have increased because LGBTQ+-identifying individuals had to spend more time in households that were not affirming of their sexual orientation or gender identity [22] and had to distance from supportive and affirming social networks [23]. Further, previous studies point to connectedness to LGBTQ + community as a protective factor between minority stress and mental health [24, 25]. Therefore, the burden of social distancing may even have a greater impact on LGBTQ + communities compared to the general population. Recent studies report that LGBTQ + people have experienced high levels of mental health problems during COVID-19-related social distancing [23, 26–29] which may be related to increased levels of social isolation, loneliness and decreased levels of social support [30]. However, only few studies [26, 28] have compared LGBTQ + people with cisgender, heterosexual individuals including rather small sample sizes. Further, recent studies also found high levels of mental health problems in the general population during COVID-19-related distancing [31–33], therefore, exploring mental health problems in people with LGBTQ + identity compared to cisgender heterosexual individuals under the conditions of severe social restriction during the lockdown at the beginning of the COVID-19 pandemic with adequate sample sizes is of scientific importance.

Therefore, the first aim of the current study was to compare mental health problems, social isolation, and loneliness during COVID-19-related social distancing between LGBTQ + identifying individuals and cisgender, heterosexual people. The second aim was to investigate whether the hypothesized higher mental health burden in LGBTQ + individuals compared to individuals not identifying as LGBTQ + is (partly) mediated by (perceived) social isolation or social support under the conditions of severe social restriction during COVID-19 lockdown. The third aim was to examine whether in-person face-to-face communication during the COVID-19 lockdown is positively associated with mental health through lower feelings of perceived social isolation, loneliness, and social support.

## Participants and methods

### Data collection

Data were collected via an online survey using LimeSurvey (LimeSurvey GmbH, Hamburg) from June 1st, 2020 until July 17th, 2020. The survey was promoted via several websites and social media platforms and through LGBTQ + organizations to also reach the LGBTQ + community. For study participation, participants had to be at least 18 years with sufficient knowledge of the German language. Participants did not receive any compensation for participating in the survey.

### Participants

Participants (N = 2369) who gave information about their gender identity and sexual orientation were included in the study. N = 531 participants indicating belonging to the LGBTQ + community filled in the survey. N = 1826 not identifying as LGBTQ + were included as a comparison group. Groups did not differ with respect to age (*t* = 0.43, *p* > .05), household income (*X*^*2*^ = 1.56, *p* > .05) and education (*X*^*2*^ = 2.53, *p* > .05). LGBTQ + persons lived significantly more often in a single household (*X*^*2*^ = 69.85, *p* < .001). Demographic characteristics can be found in Table 1.

**Table 1:** Demographics

	Non-LGBTQ+-identifying participants *N* = 1826	LGBTQ+-identifying participants *N* = 543	
**Age**			*p* > .05
Mean (SD)	42.66 (15.34)	42.35(13.76)	
Range	18–85	18–77	
**Gender identity**			
Cisman	24.0%	54.2%	
Ciswoman	76.0%	39.1%	
Transgender/queer	0%	6.6%	
**Sexual orientation**			
Bisexual	0%	31.7%	
Homosexual	0%	57.6%	
Heterosexual	100%	0.9%	
Other	0%	9.8%	
**Education level**			*p* > .05
University-level	53.2%	49.3%	
Not	46.8%	50.7%	
**Income**			*p* > .05
Low	45.4%	45.1%	
Middle	47.8%	49.6%	
High	6.9%	5.3%	
**Household**			*p* < .01
Single-household	22.0%	40.0%	
Not	78.0%	60.0%	

*Income was classified as low if the monthly net income was less than 1500 euros (below the average income in Germany), middle if the monthly net income was between 2000 and 4000 euros, and high if the monthly net income was over 4000 euros

### Measures

#### Measures of mental health

##### **Depression**

Depression was measured with the German version of the Patient Health Questionnaire (PHQ-9) [34]. The PHQ-9 scale assesses the severity of depressive symptoms with a range of 0 to 27 and a cut-off score of 10 indicating at least moderate levels of depressive symptoms.

##### **Anxiety**

Anxiety was measured with the German version of the Generalized Anxiety Disorder 7-item scale (GAD-7) [35]. The GAD-7 scale assesses the severity of generalized anxiety disorder with a maximum score of 21. A cut-off score of 10 has been shown to identify cases of moderate generalized anxiety disorder.

##### **Suicidality**

Suicidal ideation during the lockdown was captured by a question based on the third item of the German version of the Suicide Behaviours Questionnaire-Revised (SBQ-R) [36], which is acknowledged as a reliable instrument to assess suicidal ideation “How often have you thought about killing yourself during the lockdown” on a 5-point scale ranging from 1 (never) to 5 (at least 5–6 times). A dichotomous variable was created indicating whether people had suicidal thoughts during the lockdown or not.

#### Measures of social connectedness

##### **Loneliness**

Loneliness was assessed by the 11-item De Jong Gierveld Loneliness Scale, which can be applied as a unidimensional loneliness scale including items on emotional loneliness (i.e., the absence of intimate relationships) and social loneliness (i.e., the absence of a broader, engaging social network). The values range between 0 and 11 with higher values indicating more loneliness [37].

##### **Social support**

The level of social support was assessed with the help of the Oslo 3 Social Support Scale (OSSS-3) [38]. The OSSS-3 consists of three items that address the number of close friends, interests and concern from other people, and practical help from neighbors on a five-point scale. The score ranges from 3 to 14 with values between 3 and 8 representing low levels of social support, values between 9 and 11 indicating moderate levels of social support and values between 12 and 14 representing strong levels of social support.

##### **Perceived social isolation due to social distancing**

Participants were asked how much they felt socially isolated due to lockdown-related social distancing on a 6-point scale ranging from 1 (not at all) to 6 (very strong).

##### **Face-to-face contact**

Communication during the lockdown was measured by asking respondents ‘How did you regularly communicate with close friends and family since the beginning of the lockdown?’ (face-to-face, video, phone, messenger, mail, no contact). We computed the category ‘remote only or no contact’ for those who did not have ‘face-to-face’ contact during the pandemic.

### Statistical analysis

Data analysis was conducted using IBM SPSS Statistics 27.0. First, the issue of missing data was addressed. From N = 2369 individuals giving information about their sexual and gender identity, complete data were available for N = 2180 individuals. A non-significant Little’s MCAR test, *X*^*2*^ = 169.99, *p* > .05, revealed that the data were missing completely at random [39] with respect to the dependent variables, predictors, or mediators. Therefore, the expectation-maximization (EM) algorithm, implemented in SPSS, was used to fill in the missing values. All subsequent analyses used this imputed data set (N = 2369). All analyses were also repeated using listwise deletion revealing comparable findings. Analysis of covariance with group (LGBTQ + or non-LGBTQ+), as between-subjects factors and age, single household, and face-to-face contacts as a covariate on depression, anxiety, loneliness, perceived social isolation, and social support. Chi-square tests were used to assess whether suicidal thoughts, clinically-relevant depressive symptoms, and moderate levels of generalized anxiety were more likely to be present in LGBTQ + individuals. Further, it was examined whether face-to-face contacts during social distancing were differentially distributed between groups. Mediation analyses were conducted using the PROCESS procedure for SPSS v4.1 by Hayes [40] to explore whether the hypothesized difference in mental health burden between groups is mediated by feelings of social connectedness (.i.e. loneliness, perceived social isolation due to social distancing; social support). Further, mediation analyses were conducted to explore whether face-to-face contact affect mental health through feelings of social connectedness. Mediation analysis uses ordinary least squares regression or logistic regression yielding unstandardized path coefficients for direct and indirect effects. All analyses were based on 5000 bootstrapped samples. An indirect effect was considered significant if the 95% bias-corrected confidence interval did not include zero.

## Results

### Group comparisons

Chi-square tests showed that LGBTQ + individuals reported more often suicidal thoughts (28.2%) than individuals with non-LGBTQ + identity (11.1%) (*X*^2^ (1, *N =* 2369) = 95.39, *p* < .001) and had more often clinically relevant symptoms of depression based on PHQ-9 cut-off scores of 10 (39.6% compared to 25.6%, *X*^2^ (1, *N =* 2369) = 39.78, *p* < .001)) and at least moderate levels of clinically relevant generalized anxiety based on GAD-7 cut-off score of 10 (33.1% compared to 22.1%, *X*^2^ (1, *N =* 2369) = 27.39, *p* < .001)). Further, LGBTQ + people reported less face-to-face contact to close friends or family outside the own household compared to non-LGBTQ + identifying persons (59.1% compared to 54.1%, *X*^2^ (1, *N =* 2369) = 0.4.3, *p* < .05)).

Analyses of covariance with LGBTQ+ (LGBTQ + identifying individuals vs. non-LGBTQ + identifying individuals) as between-subjects factors on the continuous measures of depression, anxiety, and social connectedness were conducted controlling for age, single household, and face-to-face contact. A main effect of LGBTQ + controlling for the covariates was found for depressive symptoms (*F*(1, 2368) = 43.06, *p* <. 001), anxiety (*F*(1, 2368) = 40.71, *p* <. 001), loneliness (*F*(1, 2368) = 31.59, *p* <. 001) and social support (*F*(1, 2368) = 32.54, *p* <. 001) reflecting that LGBTQ + identifying individuals reported more depressive symptoms and anxiety and felt more social isolation during social distancing, reported more loneliness and less social support than individuals not identifying as LGBTQ+. The effect of LGBTQ + on perceived social isolation during social distancing did not reach significance when controlling for the covariates (*F*(1, 2368) = 3.36, *p* =. 067). Descriptive statistics can be found in Table 2.

The covariates age, single household and face-to-face contact were significantly related to the outcome variables reflecting that younger people, people living in a single-household and people without face-to face contact experienced more depressive symptoms, anxiety, loneliness, perceived social isolation and had less social support (depressive symptoms (age: *F*(1, 2368) = 75.09, *p* <. 001; single-household: *F*(1, 2368) = 30.21, *p* <. 001; face-to-face contact: *F*(1, 2368) = 25.82, *p* <. 001)), anxiety (age: *F*(1, 2368) = 60.68, *p* <. 001; single-household: *F*(1, 2368) = 7.69, *p* <. 01; face-to-face contact: *F*(1, 2368) = 22.08, *p* <. 001)), loneliness (age: *F*(1, 2368) = 9.91, *p* <. 01; single-household: *F*(1, 2368) = 83.23, *p* <. 001; face-to-face contact: *F*(1, 2368) = 61.08, *p* <. 001)), perceived social isolation during social distancing (age: *F*(1, 2368) = 50.52, *p* <. 001; single-household: *F*(1, 2368) = 28.69, *p* <. 001; face-to-face contact: *F*(1, 2368) = 25.40, *p* <. 001)) and social support (age: *F*(1, 2368) = 17.22, *p* <. 001; single-household: *F*(1, 2368) = 62.27, *p* <. 001; face-to-face contact: *F*(1, 2368) = 73.87, *p* <. 001))).

**Table 2:** Group differences in mental health and social connectedness

	Non-LGBTQ+-identifying participants N = 1826	LGBTQ+-identifying participants N = 543			
PHQ-9	6.76 (5.66)	8.96 (6.28)	*p* < .001		
PHQ-9 cut-off > 10	25.6%	39.6%	*p* < .001		
GAD-7	6.16 (5.04)	7.96 (5.58)	*p* < .001		
GAD-7 cut-off > 10	22.1%	33.1%	*p* < .001		
OSS-3	10.00 (2.19)	9.21 (2.19)	*p* < .001		
Loneliness	4.58 (2.88)	5.63 (2.91)	*p* < .001		
Perceived social isolation during lockdown	3.41 (1.40)	3.62 (1.43)	*p = .*067		
Suicidal thoughts	11.1%	28.2%	*p* < .001		
Face-to-face contact	59.1%	54.1%	*p* < .05		

*Data are mean (SD) or %. PHQ-9 assessed depressive symptoms, GAD-7 assessed general anxiety and OSS-3 assessed social support. Chi-square tests were used to compare distributions between groups. Analyses of covariance with LGBTQ+ (LGBTQ + identifying individuals vs. non-LGBTQ + identifying individuals) as between-subjects factors on the continuous measures of depression, anxiety, and social connectedness were conducted controlling for age, single household, and face-to-face contact

### Mediation analysis

**Fig. 1:**
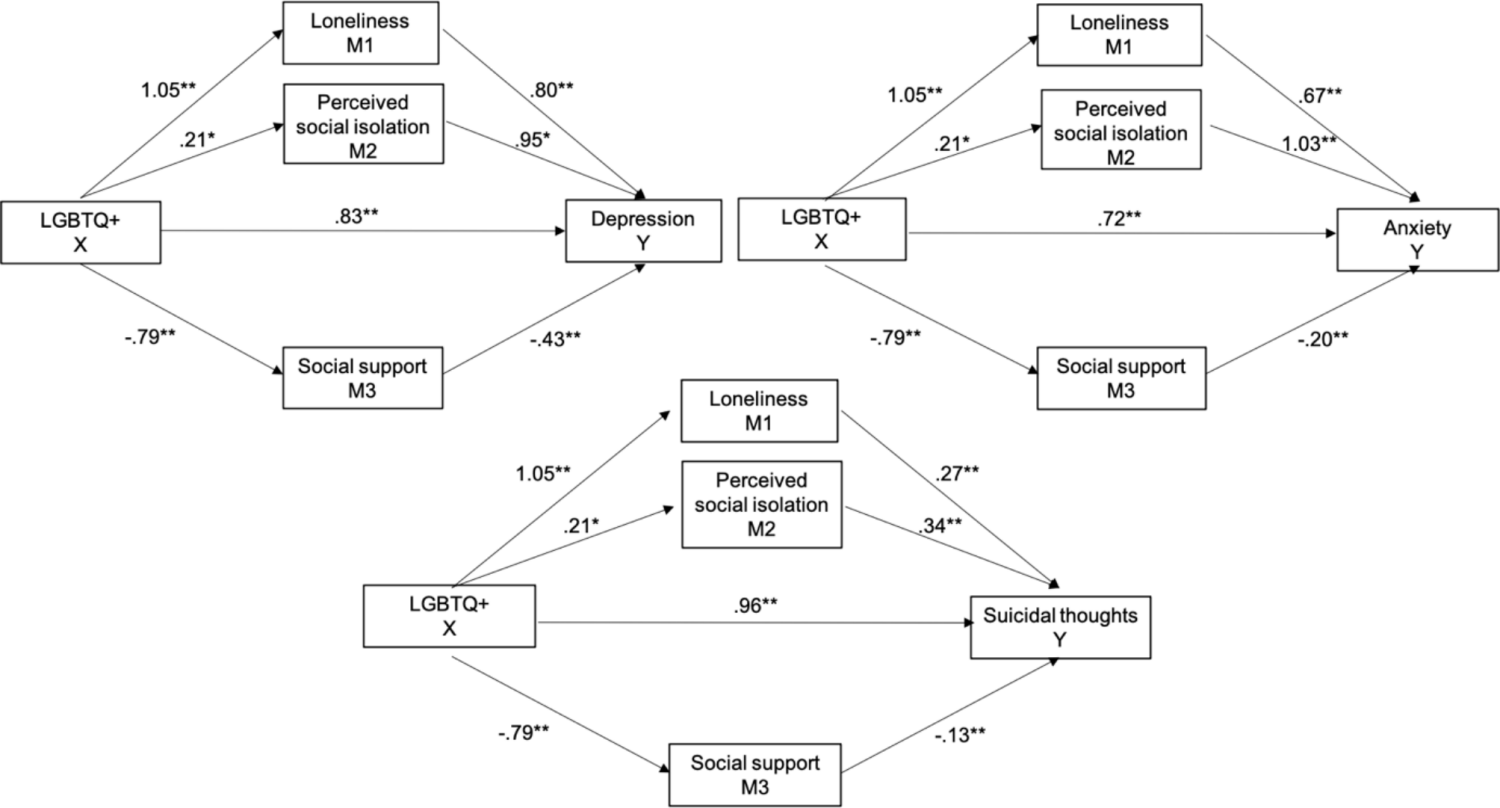
Mediation models on the impact of LGBTQ + on mental health through measures of social connectedness during the lockdown. *Unstandardized beta coefficients are presented for a’ and b’. For the direct effect (c’), the unstandardized coefficients after the mediators were added to the model are presented. The relationship between LGBTQ + identity and depression, anxiety, and suicidal thoughts was partly mediated through M1 (depression = 0.84, 95%-CI[0.60, 1.09]; anxiety = 0.71, 95%-CI[0.51, 0.92]; suicidal thoughts = 0.29 95%-CI[0.20, 0.39), M2 (depression = 0.20, 95%-CI[0.07, 0.34]; anxiety: = 0.21, 95%-CI[0.07, 0.36]; suicidal thoughts = 0.07, 95%-CI[0.02, 0.13]) and M3(depression = 0.34, 95%-CI[0.22, 0.47]; anxiety =. 16, 95%-CI[0.07, 0.27], suicidal thoughts = 0.11, 95%-CI[0.04, 0.18]). ** *p* < .01, ***p < .001

To explore whether the higher levels of depressive symptoms, anxiety, and suicidality in LGBTQ + individuals were mediated by (lack of) social connectedness, mediation analyses were run using parallel mediation models with loneliness (M1), perceived social isolation during social distancing (M2), and social support (M3) as mediators. As shown in Fig. 1, the higher levels of depression and anxiety in LGBTQ + identifying individuals (depression: direct effect = 0.83, 95%-CI[0.39, 1.27; anxiety: direct effect = 0.72, 95%-CI[0.32, 1.1]) were partly mediated through loneliness (depression: indirect effect = 0.84, 95%-CI[0.60, 1.09]; anxiety: indirect effect = 0.71, 95%-CI[0.51, 0.92]), perceived social isolation during social distancing (depression: indirect effect = 0.20, 95%-CI[0.07, 0.34]; anxiety: indirect effect = 0.21, 95%-CI[0.07, 0.36]) and social support (depression: indirect effect = 0.34, 95%-CI[0.22, 0.47]; anxiety: indirect effect = 0.16, 95%-CI[0.07, 0.27]). Further, the higher incidence of suicidal thoughts in individuals identifying as LGBTQ + compared to the comparison group (direct effect = 0.96, 95%-CI[0.69, 1.23]) was also partially mediated through loneliness (indirect effect = 0.29 95%-CI[0.20, 0.39]), perceived social isolation during social distancing (indirect effect = 0.07, 95%-CI[0.02, 0.13]) and social support (indirect effect = 0.11, 95%-CI[0.04, 0.18]) (Fig. 1).

**Fig. 2:**
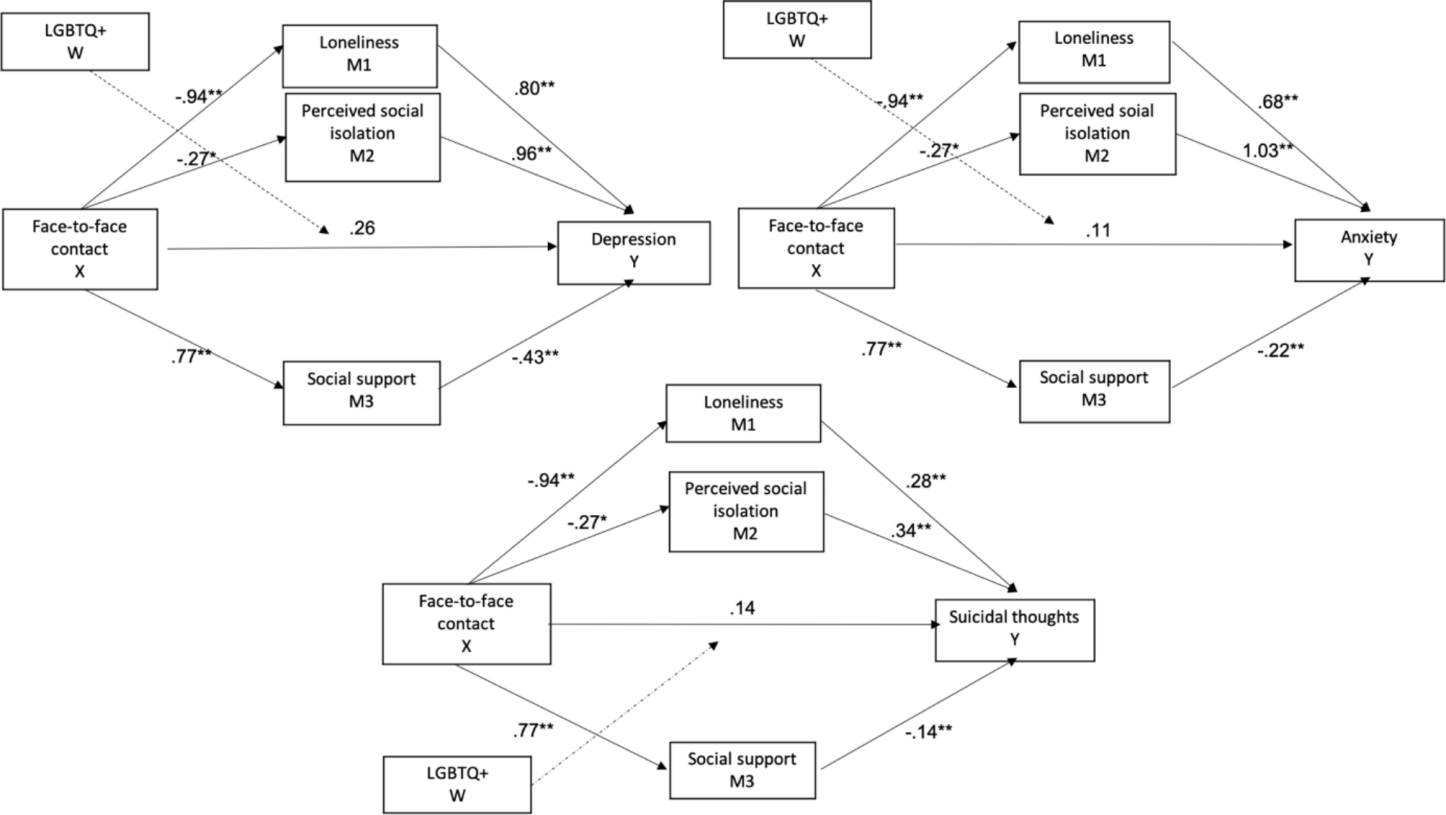
Mediation models on the impact of face-to-face contact on mental health through social connectedness during the lockdown. *Unstandardized beta coefficients are presented for a’ and b’. For the direct effect (c’), the unstandardized coefficients after the mediators were added to the model are presented. LGBTQ + identity was included as moderator indicating no significant interaction between face-to-face contact and LGBTQ + on depression (-0.21, 95%-CI[-1.07, 0.66]), anxiety (0.12, 95%-CI[-68, 0.92]) and suicidality (0.15, 95%-CI[-0.38, 0.69]).The relationship between face-to-face contact and the mental health measures was mediated by M1 (depression = .-75, 95%-CI[-0.97, − 0.55]; anxiety = − 0.63, 95%-CI[-0.81, − 0.46]; suicidality = − 0.26, 95%-CI[-0.35, − 0.18]), M2 (depression = − 0.26, 95%-CI[-0.39, − 0.15]; anxiety = − 0.28, 95%-CI[-0.41, − 0.16]; suicidality = − 0.09, 95%-CI[-0.15, − 0.05]) and M3 (depression = − 0.33, 95%-CI[-0.46, − 0.22]; anxiety = − 0.16, 95%-CI[-0.26, − 0.07]; suicidality = − 0.11, 95%-CI[-0.18, − 0.05]). ** *p* < .01, ***p < .001

Further, we wanted to explore whether in-person face-to-face communication during the lockdown affected mental health through feelings of loneliness (M1), perceived social isolation during social distancing (M2), and social support (M3). LGBTQ + identity was included as moderator revealing no significant interaction between face-to-face contact and LGBTQ + on depression (-0.21, 95%-CI[-1.07, 0.66]), anxiety (0.12, 95%-CI[-68, 0.92]) and suicidality (0.15, 95%-CI[-0.38, 0.69]), indicating that the relationship between face-to-face contact and mental health variables is not influenced by LGBTQ + identity. The effect of face-to-face contact on depression (direct effect = 0.26, 95%-CI[0.23, − 0.16]), anxiety (direct effect = 0.11, 95%-CI[-0.28, 0.51]), and suicidality (direct effect = 0.14, 95%-CI[0.85, 0.40) was mediated by feelings of loneliness (indirect effect on depression = .-75, 95%-CI[-0.97, − 0.55]; indirect effect on anxiety = − 0.63, 95%-CI[-0.81, − 0.46]; indirect effect on suicidality = − 0.26, 95%-CI[-0.35, − 0.18]), perceived social isolation during social distancing (indirect effect on depression = − 0.26, 95%-CI[-0.39, − 0.15]; indirect effect on anxiety = − 0.28, 95%-CI[-0.41, − 0.16]; indirect effect on suicidality = − 0.09, 95%-CI[-0.15, − 0.05]) and social support (indirect effect on depression = − 0.33, 95%-CI[-0.46, − 0.22]; indirect effect on anxiety = − 0.16, 95%-CI[-0.26, − 0.07]; indirect effect on suicidality = − 0.11, 95%-CI[-0.18, − 0.05]). The mediation is visualized in Fig. 2.

## Discussion

The first aim of the current study was to investigate differences in mental health and social connectedness between LGBTQ+-identifying people and people not identifying as LGBTQ+. Our findings show that depressive symptoms, anxiety, and suicidal thoughts were higher in LGBTQ+-identifying individuals than in people not identifying as LGBTQ+. 39.6% of LGBTQ+-identifying people reported at least moderate levels of depressive symptoms, 33.2% reported at least moderate levels of clinically relevant anxiety and 28.2% reported suicidal thoughts during the lockdown underlining the high mental health burden of LGBTQ + people during the lockdown. These alarming findings are in line with previous results from cross-sectional surveys during the COVID-19 pandemic describing high levels of anxiety, depression, and suicidality among the general population [41, 42] and even higher levels of anxiety, depression, and suicidality in LGBTQ + identifying individuals compared to cisgender and heterosexual individuals [23, 26–29].

Regarding social connectedness, LGBTQ + people reported more loneliness, less social support and had less face-to-face contact. Thus, individuals identifying as LGBTQ + were less socially connected than people not identifying as LGBTQ+. The differences in social isolation and loneliness between groups may be due in part to sociodemographic differences as LGBTQ + people are more likely to be childless or living alone. However, as noted in the introduction, minority stress increases social withdrawal, which may partly explain differences in social connectedness between groups [21]. Previous studies report that minority stress has increased during COVID-19 related lockdown because LGBTQ+-identifying individuals might have been “locked” with non-supportive and -affirming household members [22] and had to distance from identity affirming social networks [23]. In line with this, Kneale and Bécares [43] reported that discrimination experiences of LGBTQ + individuals predicted mental health during the pandemic.

Our second aim was to examine whether the effect of LGBTQ + on depression, anxiety, and suicidality was mediated through social connectedness. In line with our expectations, we found that loneliness, perceived social isolation during social distancing, and social support partly mediated the effect of LGBTQ + on mental health. In line with our findings, Mayerl et al. [44] showed that COVID-19-related social restrictions were associated with feelings of loneliness and predicted depressive symptoms 10 months later. Quadt et al. [45] proposed that loneliness may initiate a cascade of complex body-brain interactions responsible for severe mental and physical health problems. From an evolutionary point of view [46] social isolation may pose individuals at risk for survival and therefore the feeling of loneliness may act as an alarm signal to reconnect with others. In line with this, neuroimaging studies have shown that social connection activates reward networks [47–49] and already acute social isolation activates feelings of loneliness, which in turn, activate neuronal responses related to craving [50]. A recent study [51] provided meta-analytic evidence for the idea that loneliness up-regulates cognitive control networks to process socio-affective information, probably to reconnect with others. However, prolonged up-regulation may exhaust cognitive resources leading to difficulties in emotion regulation and thereby increasing mental health risks.

In the present study, we also found a significant effect of face-to-face communication on mental health. The moderation analysis showed that the relationship between face-to-face contact and mental health was not moderated by LGBTQ + identity, indicating that the association was comparable for both groups. Interestingly, the relationship between face-to-face contact and depression, anxiety and suicidal thoughts was (fully) mediated by feelings of loneliness, perceived social isolation during the pandemic, and social support. Thus, having no face-to-face contact during the lockdown increased feelings of loneliness, social isolation and social support, which, in turn, was negatively associated with mental health. This is in line with previous findings showing that face-to-face communication has been associated with a smaller increase in loneliness during the pandemic, which could not be found for remote-only communication [52–54]. A qualitative study during physical distancing in the initial COVID-19 lockdown in the UK described that social distancing measures impact loneliness by limiting face-to-face contact and by not perceiving digital communication as sufficient to counteract loneliness [55]. Roberts and Dunbar [56] showed that already two months of no face-to-face contact significantly reduces emotional closeness to friends. These findings suggest that social distancing may have long-lasting consequences on people’s social connectedness. This may be particularly harmful to LGBTQ + identifying people whose social network integration is particularly important to reduce minority stress and enhance identity affirmation [24, 25, 57]. Given the effects of loneliness on mental health [6], the impact of social distancing on loneliness has to be taken into account before government decisions are made on lockdown measures, and if social distancing is required, action must be taken to promote social connectedness and reduce loneliness particularly in vulnerable groups. Possible long-term consequences of social distancing on mental health should be the focus of future research, including LGBTQ + individuals who were already more vulnerable to mental health problems before the pandemic compared to heterosexual, cisgender people [12–15].

Some limitations warrant a cautious interpretation of the data. First, due to the cross-sectional design of the study, we do not have data from pre- or post-pandemic. Therefore, we do not know whether the high levels of anxiety, depression, and suicidal thoughts have already been present before the pandemic. Nevertheless, the findings show that people identifying as LGBTQ + are much more vulnerable to mental health problems compared to heterosexual, cisgender persons. Second, data was acquired in June and July of 2020 following three months of COVID-19-related lockdown in Germany. Previous studies have shown that the increase in mental health problems in the general population has decreased with the reduction of social distancing actions. However, we do not know whether this is also true for LGBTQ + identifying individuals, particularly given the high vulnerability of this population. Therefore, future studies should focus on the long-lasting consequences of social isolation for people identifying as LGBTQ + also addressing the connectedness to the LGBTQ + community, which may reduce minority stress and mental health problems [24, 25]. Third, we did not assess the frequency of face-to-face communication, which may be interesting to investigate post-pandemic to explore the role of face-to-face communication as mediating factor between LGBTQ + identity, feelings of loneliness and mental health.

## Conclusion

The current findings show that LGBTQ+-identifying individuals had significantly higher levels of depression, anxiety, and suicidal thoughts compared to individuals not identifying as LGBTQ + during the initial COVID-19-related lockdown in Germany. Interestingly, the effect of LGBTQ + on mental health was partly mediated by loneliness and social support, which, in turn, were affected by face-to-face contact during the lockdown. Given the high impact of loneliness on mental health, governmental actions should be taken to promote social connectedness particularly among LGBTQ + identifying individuals to ensure that the COVID-19 pandemic does not exacerbate the health inequalities that already exist between LGBTQ+-identifying and heterosexual, cisgender people.

## Data Availability

The dataset analyzed during the current study are available from the corresponding author on reasonable request.
